# Muscle Aging Heterogeneity: Genetic and Structural Basis of Sarcopenia Resistance

**DOI:** 10.3390/genes16080948

**Published:** 2025-08-11

**Authors:** Angelina Titova, Airat Bilyalov, Nikita Filatov, Stepan Perepechenov, Darya Kupriyanova, Sergei Brovkin, Dmitrii Shestakov, Natalia Bodunova, Oleg Gusev

**Affiliations:** 1 Institute of Fundamental Medicine and Biology, Kazan Federal University, 420008 Kazan, Russia; anjerika@list.ru (A.T.);; 2 Federal Center of Brain Research and Neurotechnology of the Federal Medical Biological Agency (FMBA) of Russia, 117513 Moscow, Russia; 3 SBHI Moscow Clinical Scientific Center Named After Loginov MHD, 111123 Moscow, Russia; 4 Life Improvement by Future Technologies (LIFT) Center, 121205 Moscow, Russia; 5 National Medical Research Centre of Cardiology Named After Academician E.I. Chazov, 121552 Moscow, Russia; 6 Intractable Disease Research Center, Graduate School of Medicine, Juntendo University, Tokyo 113-8421, Japan

**Keywords:** sarcopenia, skeletal muscle aging, muscle atrophy

## Abstract

Sarcopenia, the progressive loss of skeletal muscle mass and function with age, significantly contributes to frailty and mortality in older adults. Notably, muscles do not age uniformly—some retain structure and strength well into old age. This review explores the mechanisms underlying differential resistance to muscle aging, with a focus on sarcopenia-resistant muscles. We analyzed current literature across molecular biology, genetics, and physiology to identify key regulators of muscle preservation during aging. Special attention was given to muscle fiber types, mitochondrial function, neuromuscular junctions, and satellite cell activity. Muscles dominated by slow-twitch (type I) fibers—such as the soleus, diaphragm, and extraocular muscles—demonstrate enhanced resistance to sarcopenia. This resilience is linked to sustained oxidative metabolism, high mitochondrial density, robust antioxidant defenses, and preserved regenerative capacity. Key molecular pathways include mTOR, PGC-1α, and SIRT1/6, while genetic variants in *ACTN3*, *MSTN*, and *FOXO3* contribute to interindividual differences. In contrast, fast-twitch muscles are more vulnerable due to lower oxidative capacity and satellite cell depletion. Unique innervation patterns and neurotrophic support further protect muscles like extraocular muscles from age-related atrophy. Resistance to sarcopenia is driven by a complex interplay of intrinsic and extrinsic factors. Understanding why specific muscles age more slowly provides insights into muscle resilience and suggests novel strategies for targeted prevention and therapy. Expanding research beyond traditionally studied muscles is essential to develop comprehensive interventions to preserve mobility and independence in aging populations.

## 1. Heterogeneity in Sarcopenia Severity: Multifactorial Mechanisms Underlying Age-Related Muscle Decline

Sarcopenia is a global health problem in the present day. In the context of an overall increase in the life expectancy of the population, sarcopenia has been shown to contribute to the development of physical frailty, loss of independence, and increased mortality rates. Although muscle atrophy is a universal aspect of aging, its progression, rate, and timing can vary markedly between different cohorts of people and different muscle groups. It is noteworthy that the degree of muscle strength retention varies significantly among individuals, with some individuals demonstrating notable strength retention well into their eighth decade, while others experience a decline as early as their sixth decade of life [1]. For instance, the quadriceps muscle (rich in fast-twitch fibers) weakens faster than the soleus muscle (which is predominantly composed of slow-twitch fibers) [2]. This variability may be attributable to a multifaceted interplay of genetic predisposition, muscle fiber composition, hormonal fluctuations, and individual lifestyle factors, in addition to the presence of chronic diseases. Collectively, these factors contribute to the shaping of the trajectory of muscle aging.

Genetic factors have been posited as a contributing element to individual variability in resistance to sarcopenia. Recent studies have indicated a potential correlation between the development of sarcopenia and specific genetic polymorphisms in the methylenetetrahydrofolate reductase (*MTHFR*), alpha-actinin-3 (*ACTN3*), and nuclear respiratory factor 2 (*NRF2*) genes. The combined effects of these genetic factors may result in significant differences in muscle metabolism, protein synthesis, and response to exercise, thereby increasing or decreasing resistance to age-related loss of muscle mass [3]. A particularly noteworthy recent finding is the identification of a genetic allele of the *SIRT6* (Sirtuin 6) gene, which is a deacylase and mono-ADP ribosyltransferase (mADPr) enzyme involved in several cellular pathways that regulate aging and metabolism. The *SIRT6* gene allele has been identified in long-lived populations. This allele has been shown to exhibit increased mono-ADP-ribose isomerase (MAR) activity, which has been demonstrated to provide improved DNA repair capabilities and resistance to oxidative stress [4,5].

While genetic factors establish the baseline, modifiable factors such as low physical activity, malnutrition, and chronic disease (e.g., diabetes, COPD) accelerate the loss of muscle mass. Resistance exercise training and protein-rich diets may mitigate atrophy by stimulating mTOR and preserving type II fibers. This suggests that there is potential for personalized interventions [6]. The combination of these factors elucidates the reason why not all older adults develop sarcopenia at the same rate. A comprehensive understanding of the factors that contribute to skeletal muscle resistance is imperative for the development of more targeted preventive and therapeutic approaches aimed at preserving the health of the aging population.

The present review summarizes the current evidence on the genetic, cellular, and systemic mechanisms and factors that contribute to its slow progression or late development.

## 2. Genes Regulating Muscle Phenotype

A considerable number of studies have highlighted genetic factors as a pivotal component in the development of sarcopenia. Firstly, while skeletal myocyte metabolism follows the fundamental principles of cellular metabolism, it exhibits specialized regulatory mechanisms and adaptations that distinguish it from other somatic cell types. The regulation of muscle fiber composition, energy intake, and hypertrophic responses is dependent on the intensity and nature of the damage, as well as the degree of load in the normal state [7,8]. Several key genes can be identified that influence muscle fibers, both at the level of normal molecular regulation and dynamic adaptation.

### 2.1. The mTOR Pathway

With regard to mechanisms affecting cell survival, the mTOR (mechanistic target of rapamycin) pathway should be separately highlighted. The mTOR pathway is a fundamental regulatory axis that governs cellular growth, metabolic homeostasis, and survival mechanisms in eukaryotic systems. The mTOR pathway operates through two distinct protein complexes: mTORC1 and mTORC2, each defined by distinct substrates [6].

Recent studies have indicated that the mTOR pathway plays a pivotal role in regulating skeletal myofibril viability, exhibiting a dual capacity to both protect against sarcopenia and promote its progression, depending on the prevailing context, the balance of signaling pathways, and the employed intervention strategies. Thus, mTORC1 regulates skeletal muscle mass by balancing protein synthesis (anabolism) and breakdown (catabolism) [6,9,10,11]. mTORC1 is a multiprotein complex that functions as a central hub, integrating signals relating to growth, nutrients, and energy availability. In skeletal muscle, mTORC1 activation is required for protein accumulation and hypertrophy. The two principal mTORC1 substrates mediating substantial regulatory effects on muscle growth comprise S6K1 and 4EBP1 [12,13].

Activation of mTORC1 stimulates muscle hypertrophy through its downstream targets S6K1 and 4EBP1, enhancing protein synthesis. Mechanical loading (e.g., exercise) and nutrient availability (amino acids) serve as potent activators of this metabolic pathway [6,11,12,13]. Consistent with this, muscle-specific inactivation of the mTORC1 component raptor in muscle-specific raptor knockout (RAmKO) mice results in muscle atrophy, diminished oxidative capacity, and increased glycogen stores, ultimately leading to dystrophic alterations that are most pronounced in oxidative muscles [14]. During aging, chronic mTORC1 hyperactivation suppresses autophagy, exacerbates oxidative stress, and triggers denervation of neuromuscular junctions (NMJs), leading to muscle atrophy [15,16]. For instance, mice with genetic activation of mTORC1 (via TSC1 deletion) developed myopathy resembling sarcopenia, while rapamycin (an mTORC1 inhibitor) preserved NMJ integrity and muscle function in aged mice [17,18]. Chronic mTORC1 activation in TSC1 knockout myocytes causes mitochondrial uncoupling and excessive production of reactive oxygen species (ROS) [19,20]. Such a high-ROS environment promotes protein carbonylation and lipid peroxidation, directly damaging contractile proteins and sarcoplasmic reticulum membranes. The resulting accumulation of damaged organelles creates a self-perpetuating cycle of cellular stress that ultimately leads to fiber necrosis and replacement by fibrous tissue [19].

In TSC1 knockout mice, the muscle-specific activation of mTORC1 results in impaired neuromuscular synapse maintenance. This leads to the retraction of presynaptic terminals and the fragmentation of postsynaptic AChRs (acetylcholine receptors). The resulting pattern is reminiscent of the age-related loss of neuromuscular synapses [15,21]. Therefore, the mTORC1 signaling pathway has a dual role in sarcopenia. Under normal conditions, it supports muscle growth, but with age, its dysregulation promotes muscle loss [6,11,12,13,22]

**Figure 1:**
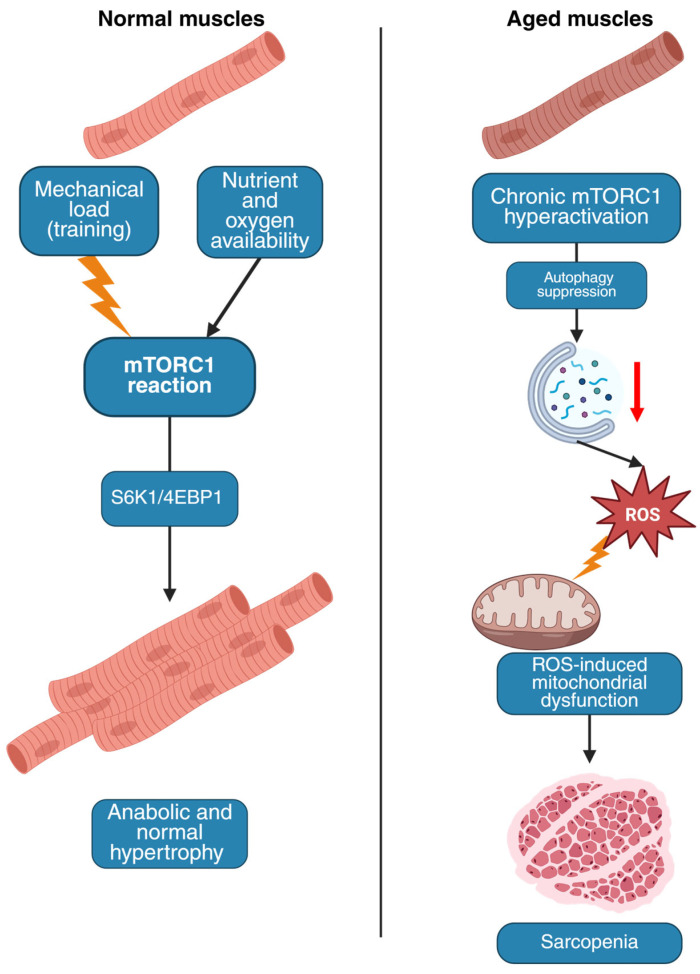
The major molecular components and signals sensed by mTORC1 and mTORC2 (in rectangles) and the processes they regulate to control cell growth and survival. The red arrow denotes a reduction in autophagic activity, while the lightning symbol represents the oxidative impact of reactive oxygen species (ROS) on mitochondrial structures.

mTORC2 exerts a unique role in skeletal muscle homeostasis, distinct from its counterpart mTORC1. While mTORC1 is well-established as a regulator of protein synthesis and degradation in sarcopenia, the effects of mTORC2 are highly conditional on the metabolic and cellular state (Figure 1) [23].

mTORC2 is essential factor in the process of Akt (Protein Kinase B, PKB) phosphorylation at ser473 (Akt Ser473). This step is considered to be a pivotal component in the complete activation of Akt for stimulation of muscle protein synthesis and suppress catabolic pathways. In addition, it has been shown to suppress nuclear translocation of *FOXO1*, thereby inhibiting the expression of atrophy-related genes such as *MuRF1* and *Atrogin* [6,24,25]. In mice with muscle-specific Rictor knockout (an essential mTORC2 component), no significant muscle atrophy was detected, indicating either the existence of compensatory mechanisms or a non-critical role of mTORC2 in maintaining muscle mass under normal physiological conditions [11,14,26]. However, mTORC2 deficiency impaired metabolic adaptation, insulin-stimulated glucose transport and reduced phosphorylation of downstream targets such as *FOXO1*, which may indirectly exacerbate sarcopenia progression [6,26,27].

It has been demonstrated that mTORC2 is implicated in the process of insulin signaling. This assertion is substantiated by experimental findings that have shown a concomitant occurrence of insulin resistance in C2C12 myotubes upon disruption of mTORC2 [28]. Moreover, mTORC2 is implicated in the thermogenic, metabolic, and growth-promoting effects induced by noradrenaline in brown adipose tissue, which indicates a potential role in metabolic regulation [29]. The observation that mTORC2-deficient mice exhibit no significant muscle pathology indicates that alternative metabolic pathways may be capable of compensating for the absence of this complex [26,30].

### 2.2. PGC-1α

**Figure 2:**
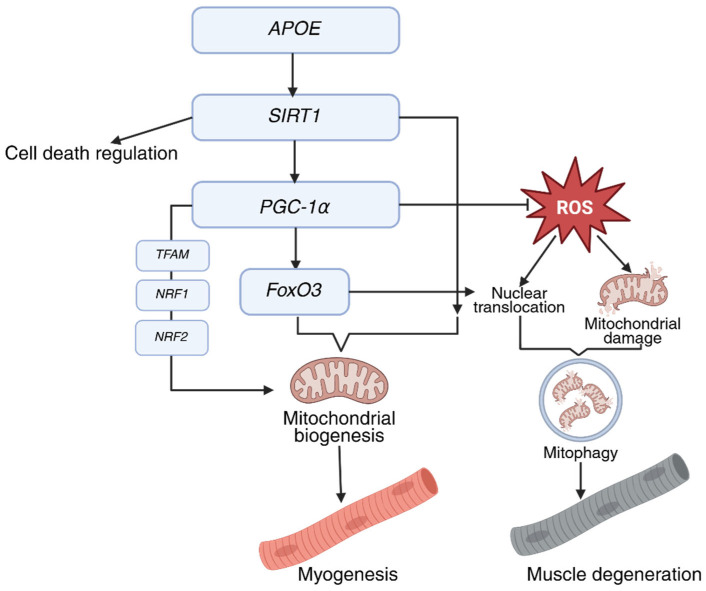
Schematic model of PGC-1α function in mitochondrial regulation and its effect on myogenesis.

Metabolic adaptations to oxidative damage in skeletal muscle fibers during prolonged aerobic exercise represent a critical determinant of muscular tissue resilience. PGC-1α regulates mitochondrial biogenesis, enhancing both mitochondrial content and the tissue’s adaptive capacity under aerobic metabolic conditions [31,32,33,34]. PGC-1α expression is highly sensitive to the metabolic demands of physical exercise, resulting in increased mitochondrial numbers and improved oxidative capacity of muscle fibers [33,35]. The result of this is an increased ability of muscle fibers to produce ATP aerobically. This, in turn, maintains sustained contractile activity and resistance to fatigue [36,37]. PGC-1α further promotes the fiber-type transition toward oxidative type I myofibers, which exhibit greater mitochondrial density and endurance capacity (Figure 2) [32,38].

PGC-1α-mediated mitochondrial biogenesis enhances the metabolic flexibility of muscle tissue by enabling efficient utilization of fatty acids and glucose as energy substrates. This is of particular significance in the context of metabolic diseases such as type 2 diabetes, in which impaired mitochondrial function contributes to insulin resistance. It has been demonstrated that physical exertion, including weight training, which has been shown to increase PGC-1α expression, can enhance glucose and lipid metabolism in muscle tissue in individuals diagnosed with diabetes. This effect is achieved through the action of the miR-30d-5p/*SIRT1*/PGC-1α axis [39,40]. Notwithstanding its pivotal function, research employing knockout models has demonstrated that PGC-1α is not exclusively accountable for mitochondrial biogenesis. The decline in mitochondrial respiratory proteins observed in PGC-1α knockout mice is minimal, with a range of 10–50%. This finding suggests the presence of compensatory mechanisms involving other transcription regulators [41]. Despite the prevalence of PGC-1α as a pivotal regulator of mitochondrial biogenesis, recent studies have indicated the involvement of other factors, including nuclear factor erythropoietin-related factor 2 (*NRF2*), estrogen-related receptor gamma (ERRγ), and peroxisome proliferator-activated receptor beta (PPARβ), in the regulation of mitochondrial content within skeletal muscle [42]. These regulators may act in concert with or independently of PGC-1α, thereby providing additional levels of regulation and potential therapeutic targets for improving mitochondrial function.

### 2.3. ACTN3

The gene known as *ACTN3*, which is also referred to as the “athlete gene”, is responsible for encoding an alpha-actinin-3 protein [43]. This protein has the function of attaching actin filaments to the Z-line within sarcomeres. It is expressed exclusively in “fast” type 2 muscle fibers [44,45]. The R577X polymorphism (rs1815739) leads to the production of either a functional protein (R allele) or a premature stop codon (X allele), which results in the complete absence of α-actinin-3 [43]. Approximately 20% of the global population is homozygous for the X allele (XX genotype) and consequently lacks α-actinin-3 in skeletal muscle tissue [46].

A number of studies have been conducted in order to examine the association between *ACTN3* genotypes and the risk of developing sarcopenia. However, the results of these studies are mixed. A study of elderly Koreans revealed a statistically significant association between genotype XX and an elevated risk of developing sarcopenia, in comparison with genotype RR [47]. In a recent study conducted on 265 elderly subjects residing in Japan, the presence of low muscle mass was found to be less prevalent amongst those who were RR homozygotes when contrasted with the X allele carriers [48]. The protective effect of the R allele against loss of muscle mass is consistent with the findings of animal studies which indicate that 18-month-old mice lacking the α-actinin-3 gene exhibit a greater decrease in muscle mass (−12.2%) compared with wild-type mice (−6.5%) [49]. A study of physically active older women found that those who were over 75 years of age and had the R allele (RR/RX genotypes) exhibited a higher propensity for developing sarcopenia in comparison to women with the XX genotype [50]. The discordant outcomes of this study imply that factors such as ethnicity, gender, physical activity level, and potentially other genetic modifiers may influence the relationship between *ACTN3* genotype and sarcopenia.

Research has demonstrated that the *ACTN3* genotype exerts an influence on the activation of the mTOR-signaling pathway in response to exercise. Individuals with the XX genotype exhibited significantly lower levels of mTOR and p70S6k phosphorylation in response to sprint training when compared to those with the RR/RX genotypes. This finding indicates that α-actinin-3 deficiency may potentially compromise the capacity of muscle tissue to initiate protein synthesis pathways in response to mechanical stimulation [51].

### 2.4. MSTN

Another key regulator of muscle metabolism is the *MSTN* (myostatin) gene. As a target of the MyoD transcription factor [52], it acts as a negative regulator of skeletal muscle growth by limiting both the number and size of muscle fibers [53] and reducing the diameter of already differentiated myofibrils [54]. Myostatin exerts concentration-dependent regulatory effects. Studies using recombinant myostatin protein in immortalized C2C12 cell lines demonstrated that at high concentrations (80–400 nM), it suppresses muscle cell proliferation [55], while at lower concentrations (2–20 nM), it conversely promotes proliferation [56]. Notably, myostatin can autoregulate its own promoter by suppressing expression through a SMAD7-dependent mechanism [53,56,57].

Mutations in the *MSTN* gene cause a 20% increase in body mass and skeletal muscle hyperplasia through elevated muscle fiber number [58]. *MSTN* demonstrates sensitivity to expression changes during muscle adaptation. In a mouse study examining gene expression during synergistic ablation-induced hypertrophy, the *MSTN* showed one of the most pronounced expression decreases genome-wide [59], highlighting its pivotal role in load-induced muscle growth regulation. As a negative regulator of muscle growth, myostatin inhibition through *MSTN* mutations may delay sarcopenia by suppressing excessive protein degradation pathways [60,61].

Despite the existence of theoretical evidence indicating the influence of specific genetic polymorphisms on muscle mass preservation, such as *MSTN* rs1805086 (affecting myostatin) and *ACTN3* rs1815739 (affecting α-actinin-3), studies conducted on Japanese centenarians have demonstrated no direct correlation between these particular variants and exceptional longevity. It is noteworthy that these populations demonstrated the highest frequency of the rs1815739 XX genotype (33.3% in male centenarians), which is associated with ‘high oxidative/efficient’ activity, suggesting potential metabolic adaptations that may indirectly support muscle maintenance [62].

## 3. Genes of Centenarians That May Affect Resistance to Sarcopenia

Centenarians exhibit distinctive genetic and molecular profiles that contribute to their exceptional longevity and reduced susceptibility to age-related diseases, including sarcopenia. While sarcopenia prevalence increases with age, studies reveal an unexpected pattern: centenarians exhibit slower and less pronounced muscle mass loss compared to younger elderly individuals [63,64,65].

*FOXO3* is the gene with the strongest and most consistent association with longevity across diverse human populations. It has been demonstrated that this gene encodes a transcription factor that plays a critical role in multiple cellular processes, including metabolism, oxidative stress response, apoptosis, and stem cell pool maintenance. Numerous studies have demonstrated that specific *FOXO3* variants are significantly overrepresented in centenarians and are linked to extended lifespan in humans [66].

In skeletal muscles, *FOXO3* functions as a pivotal transcription factor that preserves muscle homeostasis and protects against age-related atrophy. A body of research has indicated that a decline in *FOXO3* levels in the muscles of the elderly is associated with an elevated risk of sarcopenia, while the activation of *FOXO3* has been demonstrated to counteract the process of muscle aging [67]. The protective effects of *FOXO3* appear to be mediated through its regulation of key cellular processes, including protein metabolism, oxidative stress responses, and satellite cell maintenance [68,69].

In aged primates, the process of downregulation of *FOXO3* has been demonstrated to be associated with increased oxidative damage in skeletal muscle tissue [67]. *FOXO3* knockdown in C2C12 myoblasts has been demonstrated to reduce the expression of *MyoD1* and myogenin, thereby impairing myoblast differentiation into myotubes. This has been demonstrated to diminish muscle regenerative capacity, thereby exacerbating age-related atrophy [70].

Another gene potentially involved in resistance to sarcopenia is *APOE* (apolipoprotein E). The ε2 allele of the *APOE* gene is more prevalent among long-lived individuals [71,72]. Carriers of ε2 exhibit a 30–50% lower risk of muscle atrophy compared to those with the ε4 allele [71]. *APOE* ε2 has been shown to enhance lipid metabolism, reduce inflammation, and protect against oxidative stress [73,74]. In contrast, *APOE* ε4 carriers are 3.2 times more likely to have AChR antibodies, which is linked to myasthenia gravis risk, while the ε2 allele is rarely observed in such patients [75].

In lymphoblastoid cell lines derived from centenarians, *SIRT1* expression is 2.2-fold higher than in individuals aged 56–82 years (*p* = 0.001) [76]. In transgenic mice, *SIRT1* overexpression in myocytes increases the proportion of oxidative (slow-twitch) muscle fibers and enhances physical endurance [77]. In rat models of hypertension, elevated *SIRT1* levels (induced by angiotensin II receptor blockers, ARBs) promote mitochondrial biogenesis and suppress apoptosis in cardiac muscles, underscoring its protective role in striated muscle [78,79]. Furthermore, *SIRT1* inhibits NF-κB-mediated inflammation, a key driver of muscle atrophy [80].

The presented evidence suggests that resistance to sarcopenia is mediated by a complex of interrelated mechanisms, with the skeletal muscle’s ability to effectively counteract oxidative stress, maintain mitochondrial functionality, and regulate energy metabolism playing a pivotal role. As demonstrated in the relevant literature, the well-documented processes collectively constitute an integrated system that protects muscle tissue from age-related degeneration.

## 4. Mechanisms of Resistance to Sarcopenia

Skeletal muscles consist of different types of muscle fibers that vary from slow to fast in their contractile properties, which determines their functional specialization for specific tasks. Myosin heavy chain (MHC) isoforms serve as the primary marker for characterizing muscle fibers, and in most experimental approaches, the expression of corresponding MHC isoforms is used to determine muscle fiber type: slow Type I fibers (expressing MHC-I), fast Type IIa fibers (expressing MHC-IIa), and fast Type IIx fibers (expressing MHC-IIx) [81,82].

Type I fibers expressing MyHC-I are characterized predominantly by oxidative metabolism with high mitochondrial density, well-developed capillary supply, and elevated oxidative enzyme activity. Type IIa fibers represent fast oxidative-glycolytic fibers with hybrid metabolic capabilities. These fibers demonstrate moderate fatigue resistance, serving as a metabolic bridge between purely oxidative and glycolytic energy systems [83]. Type IIx fibers were traditionally characterized as fast glycolytic fibers, but modern research has revealed significant oxidative capacity in muscle fibers of this type. Studies show that Type IIx fibers can demonstrate high mitochondrial content and oxidative enzyme activity, which challenges the simple dichotomous classification of fast fibers as exclusively glycolytic. This oxidative capacity of Type IIx fibers varies significantly between different individuals and muscles, with some populations showing mitochondria-rich Type IIx fibers capable of supporting both power and endurance demands. Studies of mitochondrial content show that although Type IIx fibers typically have lower mitochondrial density compared to Type I fibers, they retain substantial oxidative capacity [84,85]. Analysis of the redox ratio shows that Type IIx fibers possess lower oxidative capacity than Type I and Type IIa fibers but are not devoid of oxidative metabolism [85,86].

Despite detailed classification of muscle fibers based on MHC isoforms, many studies still use a simplified Type I and Type II classification. Many studies are based on fundamental differences between slow oxidative (Type I) and fast glycolytic (Type II) fibers, which may be more significant for general physiological questions than subtle differences between 2A and 2X subtypes [87]. The fundamental distinction between oxidative and glycolytic characteristics captures the most physiologically and clinically relevant aspects of muscle fiber function for many applications, including exercise physiology, aging research, and disease pathology. No skeletal muscle consists exclusively of slow or fast fibers. However, muscles can be categorized based on fiber-type predominance—those with a predominantly slow or fast phenotype, as well as hybrid muscles:

1. Muscles with slow-twitch fiber predominance:
  - Soleus (maintains posture and walking);
  - Diaphragm (continuously active during respiration);
  - Spinal extensors (provide vertebral column stability).
2. Muscles with fast-twitch fiber predominance:
  - Gastrocnemius (responsible for explosive movements like jumping);
  - Biceps brachii (used for short-term forceful actions);
  - Eyelid muscles (enable rapid blinking).
3. Hybrid muscles (e.g., tibialis anterior (supports dorsiflexion and inversion of the foot, contributing to walking, running, and postural balance)) typically contain a mixed fiber composition [88].

A comparative analysis of muscle fiber types has demonstrated that the slow-twitch (Type I) phenotype exhibits distinctive metabolic adaptations that serve to mitigate sarcopenic progression. Soleus and erector spinae muscles, which are continuously engaged in postural maintenance and locomotion, exhibit sustained protein synthesis that preserves muscle mass. Research has demonstrated that chronic mechanical loading activates pathways such as mTORC1, which counteract muscle atrophy in these tissues [89,90,91,92].

Type I muscle fibers utilize aerobic metabolism (oxidative phosphorylation) rather than glycolysis, minimizing lactate accumulation and cellular stress. Oxidative phosphorylation generates 32–36 ATP per glucose molecule, compared to only 2 ATP via glycolysis, which likely reduces metabolic load during prolonged activity [90,93,94,95]. These slow oxidative fibers also express higher levels of antioxidant enzymes (e.g., superoxide dismutase, catalase, glutathione peroxidase) to neutralize ROS such as O_2_^−^ and H_2_O_2_ [96,97,98,99]. Additionally, the calcineurin/NFAT pathway—activated by calcium fluxes during contraction—promotes oxidative gene expression (e.g., myoglobin, slow troponin I) while suppressing glycolytic pathways, further reinforcing the slow-twitch phenotype and protecting against atrophy [100,101].

In contrast, fast glycolytic fibers primarily rely on anaerobic glycolysis, which generates ROS as a byproduct of rapid ATP production. Their lower antioxidant capacity makes them more susceptible to oxidative stress and damage, ultimately leading to atrophy [102,103,104,105]. These fibers also exhibit reduced levels of PGC-1α (Peroxisome Proliferator-Activated Receptor Gamma Coactivator 1-alpha), a key regulator of mitochondrial biogenesis, resulting in ROS accumulation and activation of atrophy pathways [106,107]. Furthermore, chronic ROS exposure in fast fibers activates NF-κB, triggering inflammatory cytokines and proteolytic systems (e.g., ubiquitin-proteasome), thereby promoting atrophy mechanisms [88,90].

Collectively, these mechanisms elucidate the phenomenon whereby muscles such as the soleus and postural groups demonstrate resistance to sarcopenia, whilst fast-twitch muscles (e.g., rectus femoris) undergo accelerated degeneration [89,100]. It can be posited that oxidative stress resilience is of critical importance in maintaining muscle viability during the process of aging. The transcription factor *NRF2*, a master regulator of antioxidant responses, plays a pivotal role in muscle cell biology. Research has demonstrated that *NRF2* has a positive effect on the proliferation and survival of myocytes under conditions of oxidative stress while also having the capacity to suppress the production of ROS. It is noteworthy that satellite cells exhibit a significant decrease in viability under normoxic conditions when there is an impairment of *NRF2* transcriptional activity [107].

## 5. Activity of Satellite Cells in Age-Related Atrophy

The long-term maintenance of skeletal muscle viability is contingent on satellite cells, a population of stem cells responsible for muscle tissue regeneration and repair [108]. The distribution and activity of these cells appear to vary significantly between different muscle groups, profoundly impacting both the aging process and regenerative capacity.

It is noteworthy that the masseter muscle, in contrast to other skeletal muscles, exhibits a moderate yet statistically significant increase in satellite cell density with advancing age. Conversely, the tibialis anterior and extensor hallucis longus muscles exhibit a significant depletion of these cells with advancing age [109]. These predominantly fast-twitch muscles appear to be particularly vulnerable to age-related deterioration.

In younger individuals, satellite cell distribution remains relatively balanced, with no significant difference observed between type I and II muscle fibers (0.089 ± 0.028 vs. 0.085 ± 0.033 satellite cells per fiber) [110]. However, the process of aging is associated with a significant decrease in the number of satellite cells, particularly within type II fibers. Elderly subjects exhibit significantly lower satellite cell numbers in type II fibers (0.051 ± 0.018 to 0.045 ± 0.019) compared to type I fibers (0.080 ± 0.030 to 0.081 ± 0.032) [110]. This selective depletion may drive the preferential atrophy of type II fibers, which is characteristic of sarcopenia. However, it should be noted that while satellite cell decline is associated with aging and type II fiber atrophy, current evidence does not establish a direct causal relationship between a specific (e.g., two-fold) decrease in satellite cells and the onset or progression of sarcopenia in humans [111,112]. The contribution of satellite cell loss to sarcopenia appears to be moderate and is likely one of several contributing factors.

Consequently, it can be hypothesized that the association between the process of aging and the number of satellite cells may be contingent upon the specific type of muscle in question. In addition to their primary function and involvement in regeneration, satellite cells also play a crucial role in regulating the microenvironment of muscles, in particular the extracellular matrix (ECM). Recent studies have demonstrated that satellite cells regulate ECM remodeling during the hypertrophic growth of skeletal muscles. Upon activation, satellite cells secrete exosome particles that contain microRNA-206. This microRNA then binds to the *RRBP1* mRNA in fibroblasts, thereby suppressing excessive ECM deposition [113].

This is of particular relevance to predominantly fast-twitch muscles, which undergo a loss of satellite cells with age, potentially explaining their greater susceptibility to fibrosis and impaired regeneration with age [111,114,115]. However, it is important to emphasize that, under normal aging conditions, this satellite cell decline does not lead to pathological fibrosis. The relationship between satellite cell loss and ECM changes is primarily relevant in pathological conditions involving chronic damage or disease states, rather than normal physiological aging.

**Figure 3:**
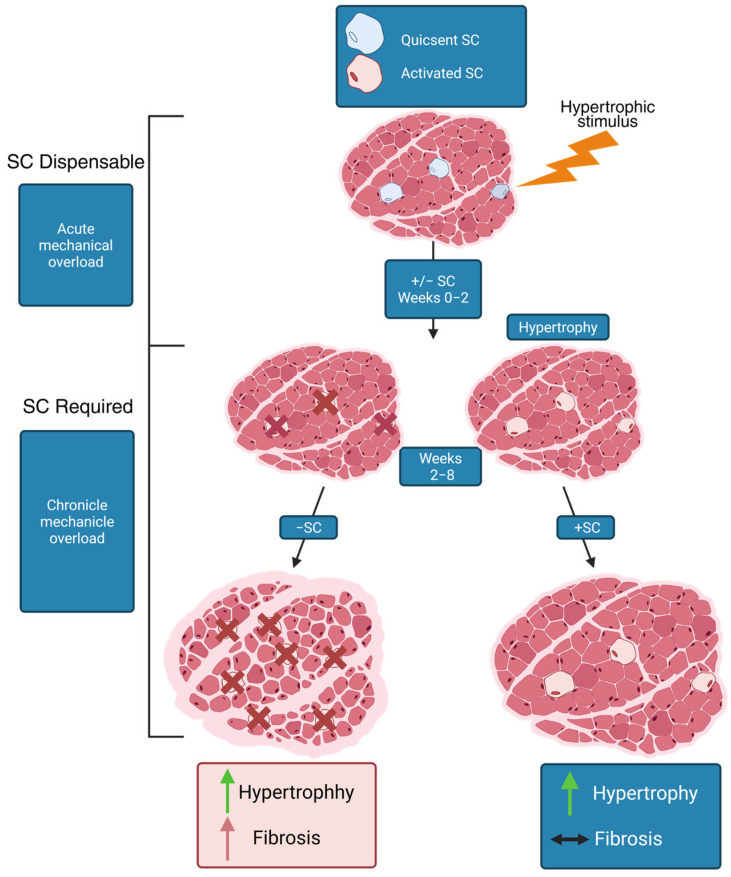
Satellite cells (SC) regulate fibrosis during hypertrophy. The green arrow signifies a positive expression of the trait, whereas the red arrow denotes a negative expression. A bidirectional black arrow the inhibition of extracellular matrix deposition, resulting in normal tissue remodeling accompanied by moderate hypertrophy. Red crosses represent cell death.

Research demonstrates that exercise by maintaining satellite cell function and promoting healthy ECM remodeling. The proliferation of satellite cells in response to hypertrophic stimuli is vital for ensuring proper remodeling of the extracellular matrix, thus preventing fibrosis, which appears to contribute to the continuation of muscle fiber hypertrophy. The absence of satellite cells has been demonstrated to result in a reduction in the rate of hypertrophy in response to prolonged mechanical stress (Figure 3) [111,114].

While the prevailing view suggests an overall age-related decline in satellite cell numbers, this trend may vary across muscle groups. Interestingly, studies on muscle regeneration in aging do not always support the assumption that slow-twitch muscles regenerate better than fast-twitch muscles with age. For example, a study examining regeneration in the fast-twitch extensor digitorum longus (EDL) muscle of mice at different ages (3-month-old young, 22-month-old aged, and 28-month-old senescent animals) found that even aged muscles retain capacity to restore their functional architecture following various injury models [115].

Unlike limb skeletal muscles, extraocular muscles (EOMs) demonstrate superior regenerative abilities and remarkable resistance to various muscular dystrophies and age-related sarcopenia. Their enhanced regenerative potential appears closely linked to their unique innervation patterns and cellular properties [116]. EOMs undergo continuous remodeling throughout life and maintain an exceptionally rich population of myogenic progenitor cells that differ both quantitatively and functionally from those in limb skeletal muscles [117,118]. Notably, EOMs show significantly higher expression of neurotrophic factors—including BDNF, NGF, NT-3, and IGF-1—compared to facial, masticatory, and limb muscles [118]. The distinctive innervation of EOMs, characterized by specialized structures like palisade endings found exclusively at myotendinous junctions, likely contributes to their enhanced regenerative capacity [119,120]. These findings suggest that innervation patterns may critically influence skeletal muscle development, function, regeneration, and resistance to age-related atrophy.

## 6. Neuronal Factors

The innervation of typical limb skeletal muscles characterized by relative uniformity, with motor neurons predominantly forming NMJs within the middle third of muscle fibers. These junctions are characterized by single innervation points per fiber and plaque-like terminals [121].

Aging induces significant alterations in this innervation pattern. Sarcopenia is a neurodegenerative process that is characterized by denervation and reinnervation, which lead to motor unit remodeling. With age, this process intensifies, significantly contributing to muscle atrophy and weakness [122]. The age-related decline in the NMJ quantity is thought to particularly affect fast-twitch muscle fibers [123]. Studies in aged female mice have demonstrated a striking increase in the percentage of completely denervated NMJs, especially in fast-contracting fibers [124]. Remarkably, the number of spinal motor neurons innervating these muscles shows no significant reduction, suggesting this may represent axonal degeneration rather than neuronal soma loss per se [125].

Recent studies have identified specific molecular regulators of NMJ integrity during aging. A significant elevation in milk fat globule–epidermal growth factor 8 (Mfge8) was observed in skeletal muscles of aged mice compared to their younger counterparts. This protein accumulates specifically at NMJs and arterial walls in aged muscle tissue [126]. Mfge8 localizes to the surfaces of end Schwann cell, which are crucial for NMJ maintenance. The upregulation of Mfge8 precedes age-related denervation and is more prominent in fast-twitch than slow-twitch muscles. The genetic deficiency of Mfge8 attenuates both age-associated NMJ denervation and muscle weakness. These findings suggest that differential Mfge8 expressions may underlie the muscle-specific susceptibility to sarcopenia, with lower expression levels potentially conferring resistance to age-related muscle atrophy [126].

Whilst the process of aging is associated with a decline in the number of motor units, there are compensatory mechanisms in place that appear to facilitate the maintenance of skeletal muscle strength. The findings of the study demonstrated that senescent rats exhibited a motor unit force output that was comparable to that of mature adult rats, despite a motor unit loss. This occurs through an increased innervation ratio (number of muscle fibers innervated by a single motor neuron), with senescent rats exhibiting approximately four-fold greater numbers of type I and IIA fibers per motor unit compared to adult rats. [127].

### Adaptive Synaptic Architecture: Extraocular Muscles and Other Examples of Stable Innervation

The EOMs, renowned for their resistance to age-related changes and pathological conditions, exhibit unique innervation patterns that distinguish them from skeletal muscles of the limbs. In EOMs, the density of nerve terminals is 10 times higher, and synapses display polymorphic structures, including en plaque endings, grape-like clusters, and spiral formations. Axons innervating phasic (twitch) muscle fibers terminate in a single NMJ, referred to in the literature as an en plaque ending. Conversely, tonic muscle fibers in the EOMs receive multiple innervations, with grape-like NMJ clusters, which are referred to in the literature as en grappe endings. This structural diversity allows for the classification of muscle fibers into singly innervated fibers (SIFs) and multiply innervated fibers (MIFs) [128]. The unique synaptic morphology of EOMs reflects their adaptation to mechanical stresses and contraction patterns. The folded structure increases synaptic surface area, enabling high-frequency acetylcholine release during rapid contractions, while spiral nerve terminals may mitigate shear forces during ocular rotations, preventing synaptic decoupling [125,128].

The molecular characteristics of EOMs, including the absence of desmin (which reduces cytoskeletal rigidity) and the retention of embryonic AChR isoforms, further support their functional stability. The persistence of fetal AChR isoforms in adult EOMs further underscores their distinct ontogenetic trajectory [124,128,129].

However, EOMs are not the only muscles with unique innervation patterns adapted to their functional demands. For instance, the thyrohyoid muscle, which forms part of the vocal apparatus, contains multiple motor endplates. Ultrastructural studies reveal that thyrohyoid muscle fibers receive dual innervation from nitrergic enteric neurons and vagal motor neurons, forming hybrid synapses. This pattern reflects the synaptic dispersion observed in EOMs [130,131,132]. The transverse and superior longitudinal muscles of the tongue similarly exhibit multiple motor endplates, analogous to EOMs, characterized by γ-AChR expression and desmin absence—features enabling precise tonic contractions essential for speech and swallowing. Like EOMs, these lingual muscles maintain embryonic AChR isoforms into adulthood, a pattern distinctly uncommon in most skeletal muscles. [128,133].

## 7. Sarcopenia-Resistant Muscles

Sarcopenia, characterized by progressive loss of muscle mass and strength, exhibits heterogeneous manifestation across different muscle groups. While limb muscles undergo significant age-related atrophy and functional decline, certain skeletal muscles maintain structural integrity and functionality even in elderly individuals. The EOMs represent the most striking example of resistance to age-related degeneration [116]. However, other muscles—including the soleus, diaphragm, tibialis anterior, and selected upper limb muscles—also demonstrate attenuated atrophy due to unique molecular, metabolic, and functional adaptations.

EOMs exhibit elevated levels of neurotrophins, including brain-derived neurotrophic factor (BDNF), nerve growth factor (NGF), and neurotrophin-3 (NT-3), which promote myoblast proliferation, fusion, and survival. These neurotrophins signal through receptors such as TrkA and p75NTR, both highly expressed in EOM progenitor cells. This unique trophic microenvironment enables EOMs to maintain remarkable regenerative capacity even during aging. BDNF/NT-3 activate mTOR pathways, enhancing protein synthesis and myofibrillar repair. p75NTR modulates pro-survival signaling, conferring protection against oxidative stress-induced apoptosis [118].

The EOMs demonstrate high mitochondrial density and unique metabolic characteristics that minimize age-associated oxidative damage. The resistance of EOMs to sarcopenia results from a combination of sustained neurotrophic support, mitochondrial robustness, enhanced oxidative stress resistance, neuromuscular synaptic redundancy, fiber-type diversity, and pro-regenerative cellular niches. These adaptive mechanisms identify potential therapeutic targets for combating age-related muscle loss and suggest promising strategies to confer EOM-like resilience to other skeletal muscles [116].

Based on 2023 review by Masatoshi Naruse (“Human Skeletal Muscle-Specific Atrophy With Aging”), we have compiled their findings into a detailed table summarizing on atrophy rates and muscle fiber type distributions (Table 1) [2,134,135].

Through analysis of EOM characteristics along with various genetic and molecular factors contributing to skeletal muscle resilience, several muscle groups emerge as particularly relevant for investigating mechanisms underlying sarcopenia resistance.

The data presented in this table demonstrate striking differences in the rate of muscle atrophy during inactivity and in age-related sarcopenia. For example, the soleus muscle exhibits the lowest rate of atrophy (0.13% per year) with aging and a high vulnerability to inactivity atrophy, which illustrates the fundamental differences between these two forms of muscle atrophy.

The accelerated atrophy observed during inactivity, compared with sarcopenia, reflects fundamentally different pathophysiological mechanisms. Atrophy of inactivity is primarily the result of a dramatic decrease in muscle protein synthesis rather than increased protein breakdown [134,135,136]. Such a rapid decrease in protein synthesis can quantify the observed loss of muscle mass during inactivity. Being the main anti-gravity muscle, the soleus muscle needs constant mechanical stress to maintain protein synthesis and structural integrity. When this stress disappears, the muscle quickly loses mechanical stimulus, leading to an immediate decrease in protein synthesis and then to a subsequent atrophy [136]. The data obtained indicate an important relationship between the composition of muscle fibers and the nature of atrophy.

Based on analysis of age-related atrophy patterns and muscle fiber type distribution, we can conclude that skeletal muscles with a predominance of slow-twitch (type I) fibers exhibit greater resistance to sarcopenia. Through comparative structural–functional analysis of lower limb muscles and extraocular muscles, we assume that it is possible to try to identify key muscle groups that appear less susceptible to age-related degeneration.

For instance, the soleus—a slow-twitch (type I) muscle—maintains mass and strength longer than adjacent muscles like the gastrocnemius [2,137]. This preservation may be attributed to its high mitochondrial density, oxidative metabolism, and continuous activation during postural maintenance. The neuromuscular stability of the soleus helps maintain motor unit integrity, thereby reducing age-related denervation [138].

It is noteworthy that the diaphragm presents another case in sarcopenia research, exhibiting unique adaptive patterns distinct from limb muscles [139]. Evidence indicates that diaphragmatic muscles demonstrate enhanced functional responsiveness to hypoxia—a phenomenon termed adaptive or compensatory plasticity. This protective mechanism mitigates oxygen deficiency and reduces detrimental effects of hypoxic stress on muscle function (hypoxic tolerance). Notably, rodent diaphragmatic muscles show relative hypoxia resistance compared to other skeletal muscles [140,141,142].

The masseter muscle contains a high proportion of type I fibers, which demonstrate oxidative stress resistance and fatigue resilience [143,144]. Compared to limb muscles, masticatory muscles maintain elevated satellite cell density, conferring superior regenerative capacity. In ALS mouse models, masseter muscles preserved normal structure and satellite cell numbers even during limb muscle atrophy. This regenerative potential may delay sarcopenic progression [145]. Aging masseter muscles exhibit elevated spermidine and valine levels versus lower limb muscles, suggesting maintained antioxidant defenses [146].

Unlike atrophy-prone gastrocnemius muscles, the tibialis anterior shows minimal mass loss during disuse. This muscle exhibits a balanced type I/II fiber composition with greater oxidative capacity than gastrocnemius [2].

Elbow flexors/extensors demonstrate moderate atrophy rates (0.38%/year) compared to lower limb muscles [2].

The multifidus muscle (MF) contains more type I fibers than erector spinae (ES). Immunofluorescence reveals significantly larger type I fiber cross-sectional area (CSA) in MF (5210 ± 1230 μm^2^) versus ES (4310 ± 980 μm^2^). These slow, oxidative fibers support MF’s role as a low-force spinal stabilizer. However, relative CSA (RCSA) shows comparable oxidative capacity (63.2% vs. 58.7%, *p* > 0.05) [147]. Despite type I fiber predominance, paraspinal muscles show no sarcopenia resistance. MF contains substantially more fibro-adipogenic progenitors (FAPs) than limb muscles, promoting adipogenic gene expression that accelerates fat infiltration and atrophy [148]. MF FAPs also overexpress αSMA versus limb FAPs, potentially creating a collagen-rich ECM that inhibits myoblast differentiation [148,149].

## 8. Conclusions

Sarcopenia represents a complex, multilevel process exhibiting significant interindividual variability and even intramuscular heterogeneity within a single organism. As demonstrated in current review, individual trajectories of muscle aging are determined by numerous factors ranging from genetic predisposition and fiber-type composition to innervation patterns, satellite cell activity, and lifestyle influences. Genetic polymorphisms in *ACTN3*, *SIRT6*, *MSTN*, and other genes establish susceptibility to either accelerated or delayed muscle mass loss. Of particular importance are cellular metabolic regulation pathways, especially mTOR- and PGC-1α-signaling cascades, which maintain the delicate balance between protein synthesis and degradation while mediating adaptation to physical activity and oxidative stress. Fiber-type composition plays a pivotal role: slow oxidative fibers (type I), predominant in the soleus and diaphragm, demonstrate remarkable sarcopenia resistance due to their tonic activation, robust antioxidant defenses, and maintained satellite cell pools. Conversely, fast glycolytic fibers (type II), characteristic of the quadriceps femoris and gastrocnemius, show greater vulnerability to age-related atrophy, attributable to their reduced oxidative stress tolerance and declining regenerative capacity with aging.

The resistance of specific muscles to sarcopenia may critically depend on their innervation patterns. Extraocular muscles, with their unique neuromuscular junction architecture and robust neurotrophic support, show remarkable resistance to age-related atrophy and serve as a model system for identifying novel therapeutic targets. The maintenance of satellite cell activity, particularly in slow-twitch muscles, enables effective regeneration and prevents fibrotic infiltration, which is crucial for preserving muscle function in aging.

Thus, sarcopenia emerges from complex interactions between genetic, cellular, metabolic, and environmental factors. Elucidating these mechanisms paves the way for developing personalized prevention and treatment strategies aimed at preserving muscle mass and functional independence in aging populations. Of particular interest are atrophy-resistant muscles—including the soleus, diaphragm, masticatory, and extraocular muscles—whose study may provide the foundation for novel therapeutic approaches against age-related muscle weakness. However, current research efforts remain significantly constrained by limited understanding of muscle-specific aging trajectories.

Current sarcopenia research faces significant limitations due to uneven scientific focus across muscle groups. A substantial proportion of all available data on age-related atrophy has been derived from studies of the quadriceps, with numerous other muscles remaining under-investigated [2]. Proteomic analyses reveal considerable differences in organelle markers, molecular chaperones, and signaling pathway expression between muscles, potentially explaining their differential atrophy susceptibility [150]. However, existing in vitro and in vivo models frequently overlook this heterogeneity by extrapolating findings from limited muscle types to the entire muscular system. Addressing these knowledge gaps requires interdisciplinary collaboration among gerontologists, biomechanists, and machine learning specialists. Only a comprehensive approach accounting for each muscle group’s unique characteristics will enable effective sarcopenia interventions and improve quality of life in aging populations.

## Figures and Tables

**Table 1:** Comparison of atrophy rates and muscle fiber types among different muscles of human. The results are presented as mean ± SD for Elbow extensors and as mean differences (MD) for the rest. *—Values represent whole muscle group averages, not individual muscles.

Muscle Group	Muscle	Atrophy Rate (%/Year) (Human) [2]	Fiber Type Composition (I/II%) Type I/Type II% (Human) [2]	Inactivity (%Atrophy Rate) (Human) [134,135]
Elbow extensors	Triceps brachii	0.38 *	36/64	5.2 ± 2.7—28 d
	Biceps brachii	0.38 *	46/54	2.8 ± 1.1—28 d
	Brachioradialis	0.38 *	44/56	n/a
Paraspinals	Erector spinae	0.47 *	61/39	n/a
	Lumbar multifidus	0.47 *	59/41	n/a
Psoas	Psoas major/Iliopsoas	0.58	45/55	2.78—14 d 4.86—28 d 2.78—42 d
Hip adductors	Adductor magnus	0.27 *	55/45	
Hamstrings	Biceps femoris short and long heads	short head 0.23 long head 0.28	54/46	1.7–2.38—3 d * 4.0–5.9—7 d * 9.3—10 d * 9.3—20 d *
	Semimembranosus	0.40	49/51	
	Semitendinosus	0.39	48/52	
Quadriceps	Rectus femoris	0.66	38/62	3.5–4.1—14 d 5.1–7.4—56 d
	Vastus lateralis	0.59	46/54	4.7–6.7—14 d * 5.6–15.9—56 d *
	Vastus intermedius	0.49	51/49	
	Vastus medialis	0.48	53/47	
Dorsiflexors	Tibialis anterior	0.19 *	74/26	0.7–9.2—14 d 0.8–7.7—28 d
Triceps surae	Gastrocnemius	0.41	59/41	Lateral: 2.4–7.7—14 d 14.4–16.5—56 d Medial: 3.0–9.4—14 d 20.4–22.3—56 d
	Soleus	0.13	81/19	3.0–9.4—14 d 20.4–22.3—56 d
